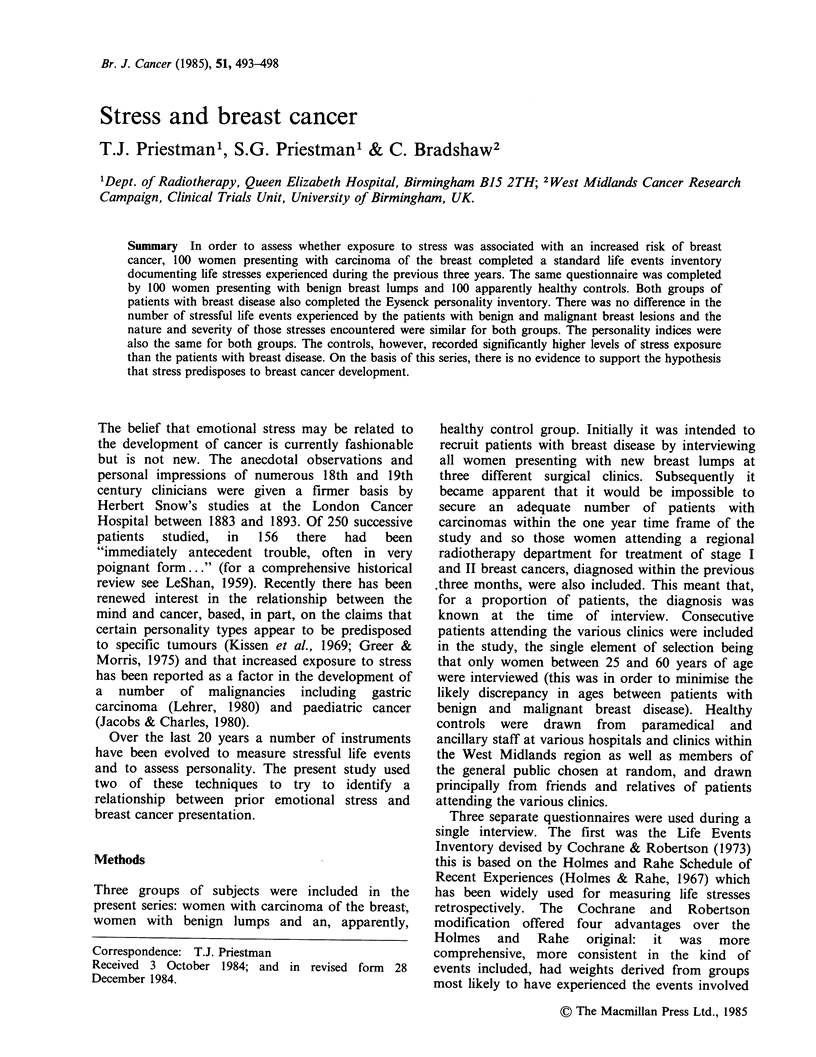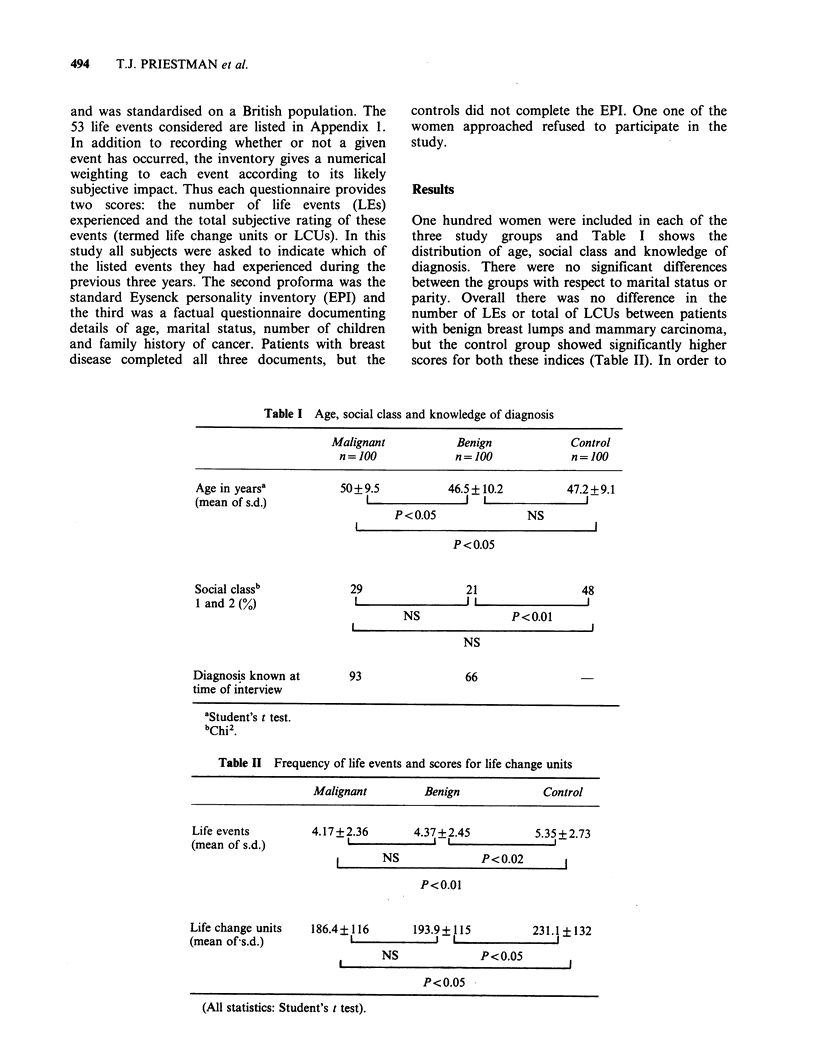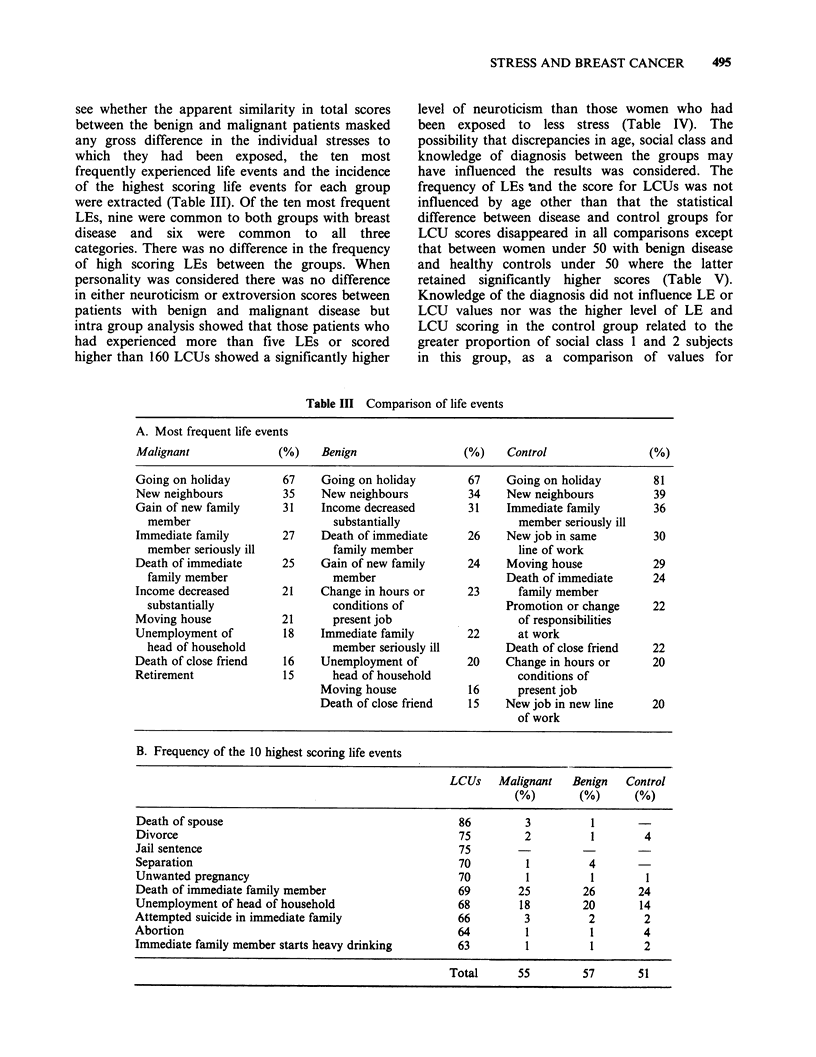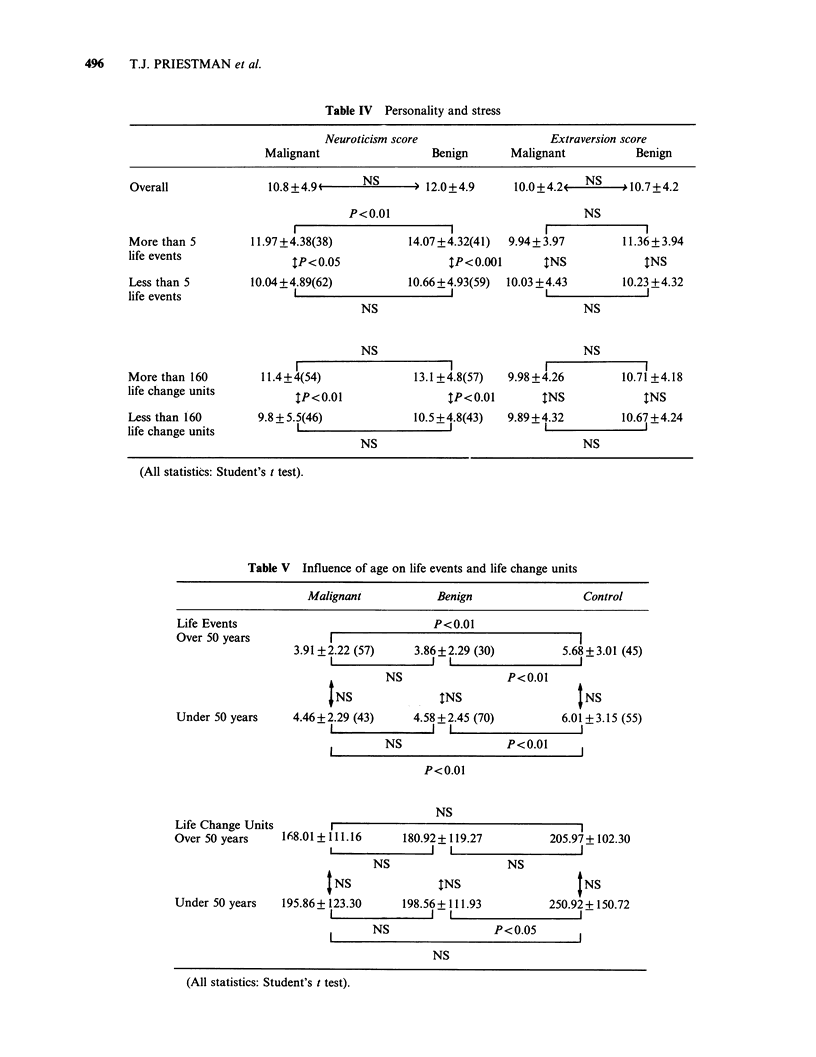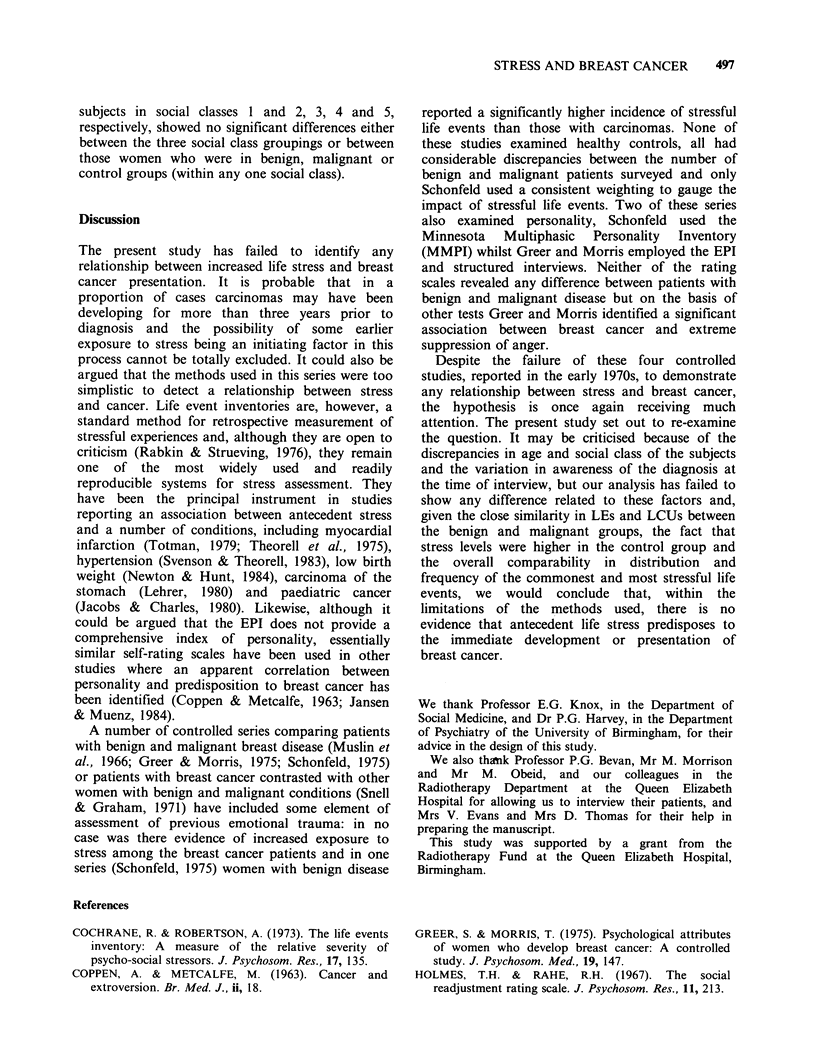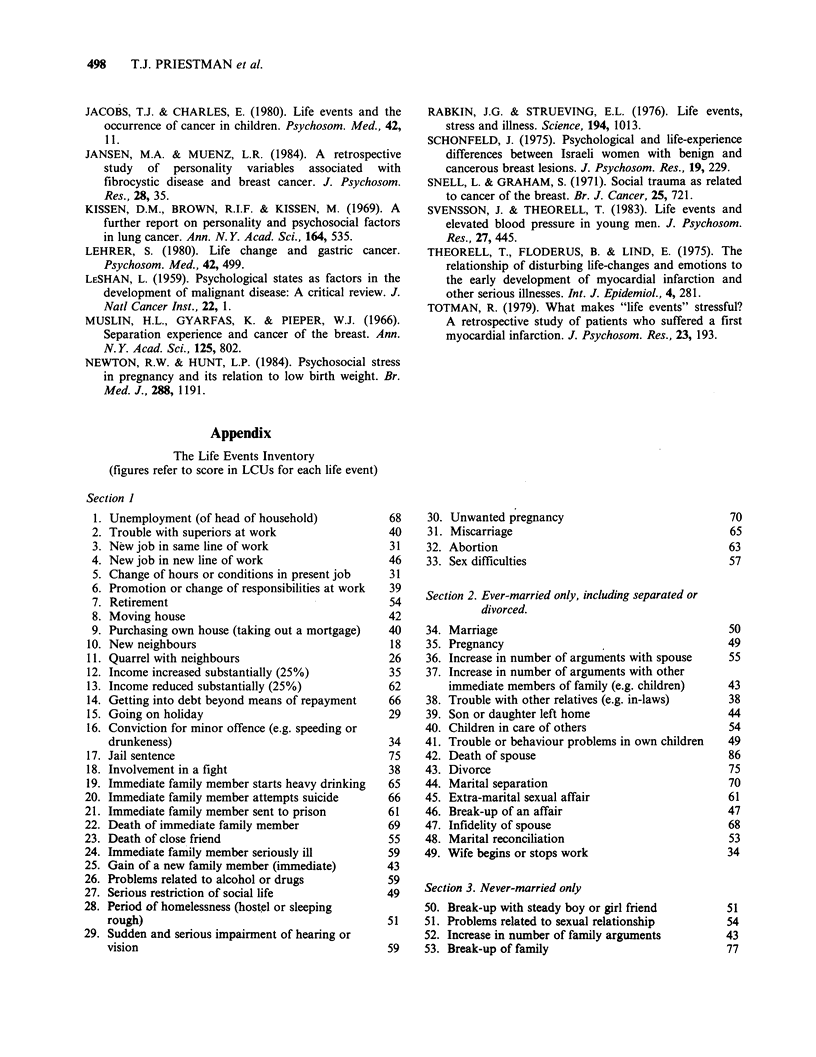# Stress and breast cancer.

**DOI:** 10.1038/bjc.1985.71

**Published:** 1985-04

**Authors:** T. J. Priestman, S. G. Priestman, C. Bradshaw

## Abstract

In order to assess whether exposure to stress was associated with an increased risk of breast cancer, 100 women presenting with carcinoma of the breast completed a standard life events inventory documenting life stresses experienced during the previous three years. The same questionnaire was completed by 100 women presenting with benign breast lumps and 100 apparently healthy controls. Both groups of patients with breast disease also completed the Eysenck personality inventory. There was no difference in the number of stressful life events experienced by the patients with benign and malignant breast lesions and the nature and severity of those stresses encountered were similar for both groups. The personality indices were also the same for both groups. The controls, however, recorded significantly higher levels of stress exposure than the patients with breast disease. On the basis of this series, there is no evidence to support the hypothesis that stress predisposes to breast cancer development.


Br. J. Cancer (1985), 51, 493–498

### Stress and breast cancer

T.J. Priestman[1], S.G. Priestman[1] & C. Bradshaw[2]

[1]*Dept. of Radiotherapy, Queen Elizabeth Hospital, Birmingham B15 2TH*; [2]*West Midlands Cancer Research Campaign, Clinical Trials Unit, University of Birmingham, UK.*

**Summary** In order to assess whether exposure to stress was associated with an increased risk of breast cancer, 100 women presenting with carcinoma of the breast completed a standard life events inventory documenting life stresses experienced during the previous three years. The same questionnaire was completed by 100 women presenting with benign breast lumps and 100 apparently healthy controls. Both groups of patients with breast disease also completed the Eysenck personality inventory. There was no difference in the number of stressful life events experienced by the patients with benign and malignant breast lesions and the nature and severity of those stresses encountered were similar for both groups. The personality indices were also the same for both groups. The controls, however, recorded significantly higher levels of stress exposure than the patients with breast disease. On the basis of this series, there is no evidence to support the hypothesis that stress predisposes to breast cancer development.

The belief that emotional stress may be related to the development of cancer is currently fashionable but is not new. The anecdotal observations and personal impressions of numerous 18th and 19th century clinicians were given a firmer basis by Herbert Snow's studies at the London Cancer Hospital between 1883 and 1893. Of 250 successive patients studied, in 156 there had been "immediately antecedent trouble, often in very poignant form..." (for a comprehensive historical review see LeShan, 1959). Recently there has been renewed interest in the relationship between the mind and cancer, based, in part, on the claims that certain personality types appear to be predisposed to specific tumours (Kissen *et al.*, 1969; Greer & Morris, 1975) and that increased exposure to stress has been reported as a factor in the development of a number of malignancies including gastric carcinoma (Lehrer, 1980) and paediatric cancer (Jacobs & Charles, 1980).

Over the last 20 years a number of instruments have been evolved to measure stressful life events and to assess personality. The present study used two of these techniques to try to identify a relationship between prior emotional stress and breast cancer presentation.

### Methods

Three groups of subjects were included in the present series: women with carcinoma of the breast, women with benign lumps and an, apparently, healthy control group. Initially it was intended to recruit patients with breast disease by interviewing all women presenting with new breast lumps at three different surgical clinics. Subsequently it became apparent that it would be impossible to secure an adequate number of patients with carcinomas within the one year time frame of the study and so those women attending a regional radiotherapy department for treatment of stage I and II breast cancers, diagnosed within the previous three months, were also included. This meant that, for a proportion of patients, the diagnosis was known at the time of interview. Consecutive patients attending the various clinics were included in the study, the single element of selection being that only women between 25 and 60 years of age were interviewed (this was in order to minimise the likely discrepancy in ages between patients with benign and malignant breast disease). Healthy controls were drawn from paramedical and ancillary staff at various hospitals and clinics within the West Midlands region as well as members of the general public chosen at random, and drawn principally from friends and relatives of patients attending the various clinics.

Three separate questionnaires were used during a single interview. The first was the Life Events Inventory devised by Cochrane & Robertson (1973) this is based on the Holmes and Rahe Schedule of Recent Experiences (Holmes & Rahe, 1967) which has been widely used for measuring life stresses retrospectively. The Cochrane and Robertson modification offered four advantages over the Holmes and Rahe original: it was more comprehensive, more consistent in the kind of events included, had weights derived from groups most likely to have experienced the events involved

Correspondence: T.J. Priestman
Received 3 October 1984; and in revised form 28 December 1984.

© The Macmillan Press Ltd., 1985

and was standardised on a British population. The 53 life events considered are listed in Appendix 1. In addition to recording whether or not a given event has occurred, the inventory gives a numerical weighting to each event according to its likely subjective impact. Thus each questionnaire provides two scores: the number of life events (LEs) experienced and the total subjective rating of these events (termed life change units or LCUs). In this study all subjects were asked to indicate which of the listed events they had experienced during the previous three years. The second proforma was the standard Eysenck personality inventory (EPI) and the third was a factual questionnaire documenting details of age, marital status, number of children and family history of cancer. Patients with breast disease completed all three documents, but the controls did not complete the EPI. One one of the women approached refused to participate in the study.

### Results

One hundred women were included in each of the three study groups and Table I shows the distribution of age, social class and knowledge of diagnosis. There were no significant differences between the groups with respect to marital status or parity. Overall there was no difference in the number of LEs or total of LCUs between patients with benign breast lumps and mammary carcinoma, but the control group showed significantly higher scores for both these indices (Table II). In order to

**Table I** Age, social class and knowledge of diagnosis

| | *Malignant* $n=100$ | *Benign* $n=100$ | *Control* $n=100$ |
|---|---|---|---|
| Age in years[a] (mean of s.d.) | 50±9.5 | 46.5±10.2 | 47.2±9.1 |
| | $P<0.05$ | NS | |
| | | $P<0.05$ | |
| Social class[b] 1 and 2 (%) | 29 | 21 | 48 |
| | NS | $P<0.01$ | |
| | | NS | |
| Diagnosis known at time of interview | 93 | 66 | — |

[a]Student's *t* test.
[b]Chi[2].

**Table II** Frequency of life events and scores for life change units

| | *Malignant* | *Benign* | *Control* |
|---|---|---|---|
| Life events (mean of s.d.) | 4.17±2.36 | 4.37±2.45 | 5.35±2.73 |
| | NS | $P<0.02$ | |
| | | $P<0.01$ | |
| Life change units (mean of s.d.) | 186.4±116 | 193.9±115 | 231.1±132 |
| | NS | $P<0.05$ | |
| | | $P<0.05$ | |

(All statistics: Student's *t* test).

see whether the apparent similarity in total scores between the benign and malignant patients masked any gross difference in the individual stresses to which they had been exposed, the ten most frequently experienced life events and the incidence of the highest scoring life events for each group were extracted (Table III). Of the ten most frequent LEs, nine were common to both groups with breast disease and six were common to all three categories. There was no difference in the frequency of high scoring LEs between the groups. When personality was considered there was no difference in either neuroticism or extroversion scores between patients with benign and malignant disease but intra group analysis showed that those patients who had experienced more than five LEs or scored higher than 160 LCUs showed a significantly higher level of neuroticism than those women who had been exposed to less stress (Table IV). The possibility that discrepancies in age, social class and knowledge of diagnosis between the groups may have influenced the results was considered. The frequency of LEs and the score for LCUs was not influenced by age other than that the statistical difference between disease and control groups for LCU scores disappeared in all comparisons except that between women under 50 with benign disease and healthy controls under 50 where the latter retained significantly higher scores (Table V). Knowledge of the diagnosis did not influence LE or LCU values nor was the higher level of LE and LCU scoring in the control group related to the greater proportion of social class 1 and 2 subjects in this group, as a comparison of values for

**Table III** Comparison of life events

A. Most frequent life events

| *Malignant* | (%) | *Benign* | (%) | *Control* | (%) |
|---|---|---|---|---|---|
| Going on holiday | 67 | Going on holiday | 67 | Going on holiday | 81 |
| New neighbours | 35 | New neighbours | 34 | New neighbours | 39 |
| Gain of new family member | 31 | Income decreased substantially | 31 | Immediate family member seriously ill | 36 |
| Immediate family member seriously ill | 27 | Death of immediate family member | 26 | New job in same line of work | 30 |
| Death of immediate family member | 25 | Gain of new family member | 24 | Moving house | 29 |
| Income decreased substantially | 21 | Change in hours or conditions of present job | 23 | Death of immediate family member | 24 |
| Moving house | 21 | Immediate family member seriously ill | 22 | Promotion or change of responsibilities at work | 22 |
| Unemployment of head of household | 18 | Unemployment of head of household | 20 | Death of close friend | 22 |
| Death of close friend | 16 | Moving house | 16 | Change in hours or conditions of present job | 20 |
| Retirement | 15 | Death of close friend | 15 | New job in new line of work | 20 |

B. Frequency of the 10 highest scoring life events

| | *LCUs* | *Malignant* (%) | *Benign* (%) | *Control* (%) |
|---|---|---|---|---|
| Death of spouse | 86 | 3 | 1 | — |
| Divorce | 75 | 2 | 1 | 4 |
| Jail sentence | 75 | — | — | — |
| Separation | 70 | 1 | 4 | — |
| Unwanted pregnancy | 70 | 1 | 1 | 1 |
| Death of immediate family member | 69 | 25 | 26 | 24 |
| Unemployment of head of household | 68 | 18 | 20 | 14 |
| Attempted suicide in immediate family | 66 | 3 | 2 | 2 |
| Abortion | 64 | 1 | 1 | 4 |
| Immediate family member starts heavy drinking | 63 | 1 | 1 | 2 |
| | Total | 55 | 57 | 51 |

**Table IV** Personality and stress

| | *Neuroticism score* Malignant | *Neuroticism score* Benign | *Extraversion score* Malignant | *Extraversion score* Benign |
|---|---|---|---|---|
| Overall | 10.8 ± 4.9 ←NS→ | 12.0 ± 4.9 | 10.0 ± 4.2 ←NS→ | 10.7 ± 4.2 |
| | $P<0.01$ (Malignant vs Benign) | | NS (Malignant vs Benign) | |
| More than 5 life events | 11.97 ± 4.38(38) | 14.07 ± 4.32(41) | 9.94 ± 3.97 | 11.36 ± 3.94 |
| | ↕$P<0.05$ | ↕$P<0.001$ | ↕NS | ↕NS |
| Less than 5 life events | 10.04 ± 4.89(62) | 10.66 ± 4.93(59) | 10.03 ± 4.43 | 10.23 ± 4.32 |
| | NS (Malignant vs Benign) | | NS (Malignant vs Benign) | |
| | NS (Malignant vs Benign) | | NS (Malignant vs Benign) | |
| More than 160 life change units | 11.4 ± 4(54) | 13.1 ± 4.8(57) | 9.98 ± 4.26 | 10.71 ± 4.18 |
| | ↕$P<0.01$ | ↕$P<0.01$ | ↕NS | ↕NS |
| Less than 160 life change units | 9.8 ± 5.5(46) | 10.5 ± 4.8(43) | 9.89 ± 4.32 | 10.67 ± 4.24 |
| | NS (Malignant vs Benign) | | NS (Malignant vs Benign) | |

(All statistics: Student's *t* test).

**Table V** Influence of age on life events and life change units

| | *Malignant* | *Benign* | *Control* |
|---|---|---|---|
| **Life Events** | | | |
| | $P<0.01$ (Malignant vs Control) | | |
| Over 50 years | 3.91 ± 2.22 (57) | 3.86 ± 2.29 (30) | 5.68 ± 3.01 (45) |
| | NS (Malignant vs Benign) | $P<0.01$ (Benign vs Control) | |
| | ↕NS | ↕NS | ↕NS |
| Under 50 years | 4.46 ± 2.29 (43) | 4.58 ± 2.45 (70) | 6.01 ± 3.15 (55) |
| | NS (Malignant vs Benign) | $P<0.01$ (Benign vs Control) | |
| | $P<0.01$ (Malignant vs Control) | | |
| **Life Change Units** | | | |
| | NS (Malignant vs Control) | | |
| Over 50 years | 168.01 ± 111.16 | 180.92 ± 119.27 | 205.97 ± 102.30 |
| | NS (Malignant vs Benign) | NS (Benign vs Control) | |
| | ↕NS | ↕NS | ↕NS |
| Under 50 years | 195.86 ± 123.30 | 198.56 ± 111.93 | 250.92 ± 150.72 |
| | NS (Malignant vs Benign) | $P<0.05$ (Benign vs Control) | |
| | NS (Malignant vs Control) | | |

(All statistics: Student's *t* test).

subjects in social classes 1 and 2, 3, 4 and 5, respectively, showed no significant differences either between the three social class groupings or between those women who were in benign, malignant or control groups (within any one social class).

### Discussion

The present study has failed to identify any relationship between increased life stress and breast cancer presentation. It is probable that in a proportion of cases carcinomas may have been developing for more than three years prior to diagnosis and the possibility of some earlier exposure to stress being an initiating factor in this process cannot be totally excluded. It could also be argued that the methods used in this series were too simplistic to detect a relationship between stress and cancer. Life event inventories are, however, a standard method for retrospective measurement of stressful experiences and, although they are open to criticism (Rabkin & Strueving, 1976), they remain one of the most widely used and readily reproducible systems for stress assessment. They have been the principal instrument in studies reporting an association between antecedent stress and a number of conditions, including myocardial infarction (Totman, 1979; Theorell *et al.*, 1975), hypertension (Svenson & Theorell, 1983), low birth weight (Newton & Hunt, 1984), carcinoma of the stomach (Lehrer, 1980) and paediatric cancer (Jacobs & Charles, 1980). Likewise, although it could be argued that the EPI does not provide a comprehensive index of personality, essentially similar self-rating scales have been used in other studies where an apparent correlation between personality and predisposition to breast cancer has been identified (Coppen & Metcalfe, 1963; Jansen & Muenz, 1984).

A number of controlled series comparing patients with benign and malignant breast disease (Muslin *et al.*, 1966; Greer & Morris, 1975; Schonfeld, 1975) or patients with breast cancer contrasted with other women with benign and malignant conditions (Snell & Graham, 1971) have included some element of assessment of previous emotional trauma: in no case was there evidence of increased exposure to stress among the breast cancer patients and in one series (Schonfeld, 1975) women with benign disease reported a significantly higher incidence of stressful life events than those with carcinomas. None of these studies examined healthy controls, all had considerable discrepancies between the number of benign and malignant patients surveyed and only Schonfeld used a consistent weighting to gauge the impact of stressful life events. Two of these series also examined personality, Schonfeld used the Minnesota Multiphasic Personality Inventory (MMPI) whilst Greer and Morris employed the EPI and structured interviews. Neither of the rating scales revealed any difference between patients with benign and malignant disease but on the basis of other tests Greer and Morris identified a significant association between breast cancer and extreme suppression of anger.

Despite the failure of these four controlled studies, reported in the early 1970s, to demonstrate any relationship between stress and breast cancer, the hypothesis is once again receiving much attention. The present study set out to re-examine the question. It may be criticised because of the discrepancies in age and social class of the subjects and the variation in awareness of the diagnosis at the time of interview, but our analysis has failed to show any difference related to these factors and, given the close similarity in LEs and LCUs between the benign and malignant groups, the fact that stress levels were higher in the control group and the overall comparability in distribution and frequency of the commonest and most stressful life events, we would conclude that, within the limitations of the methods used, there is no evidence that antecedent life stress predisposes to the immediate development or presentation of breast cancer.

We thank Professor E.G. Knox, in the Department of Social Medicine, and Dr P.G. Harvey, in the Department of Psychiatry of the University of Birmingham, for their advice in the design of this study.

We also thank Professor P.G. Bevan, Mr M. Morrison and Mr M. Obeid, and our colleagues in the Radiotherapy Department at the Queen Elizabeth Hospital for allowing us to interview their patients, and Mrs V. Evans and Mrs D. Thomas for their help in preparing the manuscript.

This study was supported by a grant from the Radiotherapy Fund at the Queen Elizabeth Hospital, Birmingham.

### References

COCHRANE, R. & ROBERTSON, A. (1973). The life events inventory: A measure of the relative severity of psycho-social stressors. *J. Psychosom. Res.*, **17**, 135.

COPPEN, A. & METCALFE, M. (1963). Cancer and extroversion. *Br. Med. J.*, **ii**, 18.

GREER, S. & MORRIS, T. (1975). Psychological attributes of women who develop breast cancer: A controlled study. *J. Psychosom. Med.*, **19**, 147.

HOLMES, T.H. & RAHE, R.H. (1967). The social readjustment rating scale. *J. Psychosom. Res.*, **11**, 213.

JACOBS, T.J. & CHARLES, E. (1980). Life events and the occurrence of cancer in children. *Psychosom. Med.*, **42**, 11.

JANSEN, M.A. & MUENZ, L.R. (1984). A retrospective study of personality variables associated with fibrocystic disease and breast cancer. *J. Psychosom. Res.*, **28**, 35.

KISSEN, D.M., BROWN, R.I.F. & KISSEN, M. (1969). A further report on personality and psychosocial factors in lung cancer. *Ann. N.Y. Acad. Sci.*, **164**, 535.

LEHRER, S. (1980). Life change and gastric cancer. *Psychosom. Med.*, **42**, 499.

LeSHAN, L. (1959). Psychological states as factors in the development of malignant disease: A critical review. *J. Natl Cancer Inst.*, **22**, 1.

MUSLIN, H.L., GYARFAS, K. & PIEPER, W.J. (1966). Separation experience and cancer of the breast. *Ann. N.Y. Acad. Sci.*, **125**, 802.

NEWTON, R.W. & HUNT, L.P. (1984). Psychosocial stress in pregnancy and its relation to low birth weight. *Br. Med. J.*, **288**, 1191.

RABKIN, J.G. & STRUEVING, E.L. (1976). Life events, stress and illness. *Science*, **194**, 1013.

SCHONFELD, J. (1975). Psychological and life-experience differences between Israeli women with benign and cancerous breast lesions. *J. Psychosom. Res.*, **19**, 229.

SNELL, L. & GRAHAM, S. (1971). Social trauma as related to cancer of the breast. *Br. J. Cancer*, **25**, 721.

SVENSSON, J. & THEORELL, T. (1983). Life events and elevated blood pressure in young men. *J. Psychosom. Res.*, **27**, 445.

THEORELL, T., FLODERUS, B. & LIND, E. (1975). The relationship of disturbing life-changes and emotions to the early development of myocardial infarction and other serious illnesses. *Int. J. Epidemiol.*, **4**, 281.

TOTMAN, R. (1979). What makes "life events" stressful? A retrospective study of patients who suffered a first myocardial infarction. *J. Psychosom. Res.*, **23**, 193.

### Appendix

The Life Events Inventory
(figures refer to score in LCUs for each life event)

*Section 1*

| | | |
|---|---|---|
| 1. | Unemployment (of head of household) | 68 |
| 2. | Trouble with superiors at work | 40 |
| 3. | New job in same line of work | 31 |
| 4. | New job in new line of work | 46 |
| 5. | Change of hours or conditions in present job | 31 |
| 6. | Promotion or change of responsibilities at work | 39 |
| 7. | Retirement | 54 |
| 8. | Moving house | 42 |
| 9. | Purchasing own house (taking out a mortgage) | 40 |
| 10. | New neighbours | 18 |
| 11. | Quarrel with neighbours | 26 |
| 12. | Income increased substantially (25%) | 35 |
| 13. | Income reduced substantially (25%) | 62 |
| 14. | Getting into debt beyond means of repayment | 66 |
| 15. | Going on holiday | 29 |
| 16. | Conviction for minor offence (e.g. speeding or drunkeness) | 34 |
| 17. | Jail sentence | 75 |
| 18. | Involvement in a fight | 38 |
| 19. | Immediate family member starts heavy drinking | 65 |
| 20. | Immediate family member attempts suicide | 66 |
| 21. | Immediate family member sent to prison | 61 |
| 22. | Death of immediate family member | 69 |
| 23. | Death of close friend | 55 |
| 24. | Immediate family member seriously ill | 59 |
| 25. | Gain of a new family member (immediate) | 43 |
| 26. | Problems related to alcohol or drugs | 59 |
| 27. | Serious restriction of social life | 49 |
| 28. | Period of homelessness (hostel or sleeping rough) | 51 |
| 29. | Sudden and serious impairment of hearing or vision | 59 |
| 30. | Unwanted pregnancy | 70 |
| 31. | Miscarriage | 65 |
| 32. | Abortion | 63 |
| 33. | Sex difficulties | 57 |

*Section 2. Ever-married only, including separated or divorced.*

| | | |
|---|---|---|
| 34. | Marriage | 50 |
| 35. | Pregnancy | 49 |
| 36. | Increase in number of arguments with spouse | 55 |
| 37. | Increase in number of arguments with other immediate members of family (e.g. children) | 43 |
| 38. | Trouble with other relatives (e.g. in-laws) | 38 |
| 39. | Son or daughter left home | 44 |
| 40. | Children in care of others | 54 |
| 41. | Trouble or behaviour problems in own children | 49 |
| 42. | Death of spouse | 86 |
| 43. | Divorce | 75 |
| 44. | Marital separation | 70 |
| 45. | Extra-marital sexual affair | 61 |
| 46. | Break-up of an affair | 47 |
| 47. | Infidelity of spouse | 68 |
| 48. | Marital reconciliation | 53 |
| 49. | Wife begins or stops work | 34 |

*Section 3. Never-married only*

| | | |
|---|---|---|
| 50. | Break-up with steady boy or girl friend | 51 |
| 51. | Problems related to sexual relationship | 54 |
| 52. | Increase in number of family arguments | 43 |
| 53. | Break-up of family | 77 |